# Auditory EEG Biomarkers in Fragile X Syndrome: Clinical Relevance

**DOI:** 10.3389/fnint.2019.00060

**Published:** 2019-10-09

**Authors:** Lauren E. Ethridge, Lisa A. De Stefano, Lauren M. Schmitt, Nicholas E. Woodruff, Kara L. Brown, Morgan Tran, Jun Wang, Ernest V. Pedapati, Craig A. Erickson, John A. Sweeney

**Affiliations:** ^1^Department of Pediatrics, Section of Developmental and Behavioral Pediatrics, The University of Oklahoma Health Sciences Center, Oklahoma City, OK, United States; ^2^Department of Psychology, The University of Oklahoma, Norman, OK, United States; ^3^Division of Developmental and Behavioral Pediatrics, Cincinnati Children’s Hospital Medical Center, Cincinnati, OH, United States; ^4^Department of Psychiatry and Behavioral Neuroscience, University of Cincinnati, Cincinnati, OH, United States; ^5^Department of Psychology, Zhejiang Normal University, Jinhua, China; ^6^Division of Child and Adolescent Psychiatry, Cincinnati Children’s Hospital Medical Center, Cincinnati, OH, United States; ^7^Division of Child Neurology, Cincinnati Children’s Hospital Medical Center, Cincinnati, OH, United States

**Keywords:** Fragile X Syndrome, EEG, chirp, habituation, gamma, sensory

## Abstract

Sensory hypersensitivities are common and distressing features of Fragile X Syndrome (FXS). While there are many drug interventions that reduce behavioral deficits in Fmr1 mice and efforts to translate these preclinical breakthroughs into clinical trials for FXS, evidence-based clinical interventions are almost non-existent potentially due to lack of valid neural biomarkers. Local circuit function in sensory networks is dependent on the dynamic balance of activity in inhibitory/excitatory synapses. Studies are needed to examine the association of electrophysiological alterations in neural systems with sensory and other clinical features of FXS to establish their clinical relevance. Adolescents and adults with FXS (*n* = 38, Mean age = 25.5, std = 10.1; 13 females) and age matched typically developing controls (*n* = 40, Mean age = 27.7, std = 12.1; 17 females) completed auditory chirp and auditory habituation tasks while undergoing dense array electroencephalography (EEG). Amplitude, latency, and percent change (habituation) in N1 and P2 event-related potential (ERP) components were characterized for the habituation task; time-frequency calculations using Morlet wavelets characterized phase-locking and single trial power for the habituation and chirp tasks. FXS patients showed increased amplitude but some evidence for reduced habituation of the N1 ERP, and reduced phase-locking in the low and high gamma frequency range and increased low gamma power to the chirp stimulus. FXS showed increased theta power in both tasks. While the habituation finding was weaker than previously found, the remaining findings replicate our previous work in a new sample of patients with FXS. Females showed less deficit in the chirp task but not the habituation task. Abnormal increases in gamma power were related to more severe behavioral and psychiatric features as well as reductions in neurocognitive abilities. Replicating electrophysiological deficits in a new group of patients using different EEG equipment at a new data collection site with differing levels of environmental noise that were robust to data processing techniques utilizing multiple researchers, indicates a potential for scalability to multi-site clinical trials. Given the robust replicability, relevance to clinical measures, and preclinical evidence for sensitivity of these EEG measures to pharmacological intervention, the observed abnormalities may provide novel translational markers of target engagement and potentially outcome measures in large-scale studies evaluating new treatments targeting neural hyperexcitability in FXS.

## Introduction

While there are many drug interventions that reduce behavioral deficits in Fmr1 mice and efforts to translate these preclinical breakthroughs into clinical trials for Fragile X Syndrome (FXS) (Tranfaglia, 2011; Wijetunge et al., 2013; Budimirovic et al., 2017; Berry-Kravis et al., 2018; Erickson et al., 2018), evidence-based clinical interventions are almost non-existent (Berry-Kravis et al., 2018). One advance that may speed progress in treatment development is the establishment of valid biomarkers of brain activity that can be used to stratify patients based on presence of abnormalities targeted by novel drugs (Erickson et al., 2018).

Auditory hypersensitivities and other sensory processing abnormalities are common in FXS as well as idiopathic autism spectrum disorder (ASD) (Takarae et al., 2007, 2014; Hagerman, 2008; Hall et al., 2009; Matsuzaki et al., 2012; Ethridge et al., 2017). Our previous electroencephalography (EEG) and event-related potential (ERP) studies demonstrated electrophysiological phenotypes that show considerable conservation across Fmr1 knock-out (KO) mice and FXS patients, indicating that they may represent promising biomarkers for FXS. However, replication in a larger independent patient population, evaluation of clinical correlates, addressing specificity, and evaluation of scalability considerations in data collection are needed to further validate these evoked EEG measures.

Our previous findings showed significantly increased non-specific gamma activity (gamma single-trial power) in FXS that was associated with a decreased ability to (1) transiently synchronize evoked gamma (the “gamma spike” during early stimulus registration), (2) to synchronize evoked gamma to a rapidly changing oscillatory “chirp” stimulus (Ethridge et al., 2017) and (3) to habituate the neural response to repeated tones (Ethridge et al., 2016). These abnormalities were associated with increased clinical measures of sensory hypersensitivity, suggesting altered gamma oscillations/neural hyper-excitability are a potential biomarker of sensory issues in FXS. Still whether this potential biomarker has clinical relevance beyond sensory issues, including links to cardinal behavioral and cognitive features, remains unknown.

Gamma band activity has established neural mechanisms, which include the local circuit glutamate/GABA interactions involving excitation onto and inhibition originating from parvalbumin positive (PV+) fast-spiking interneurons (the PING model), and mutually connected inhibitory interneurons (the ING model) (Gibson et al., 2008; Cardin et al., 2009; Tiesinga and Sejnowski, 2009). During sensory processing gamma is associated with bottom-up sensory processing of basic stimulus properties (Brosch et al., 2002). Reduced local circuit inhibition via the PING model has been proposed as a neural mechanism for sensory hypersensitivity and neural hyper-excitability in FXS (Gibson et al., 2008; Paluszkiewicz et al., 2011). Importantly, these neural phenotypes have been largely replicated in Fmr1 KO mice, including increased gamma power and abnormal synchronization at both the *in vivo* (Sinclair et al., 2017; Lovelace et al., 2018) and *in vitro* (Goswami et al., 2019) levels. Gamma power and synchronization abnormalities also show preclinical responsiveness to both genetic (Wen et al., 2018) and pharmaceutical (Sinclair et al., 2017; Lovelace et al., 2018) intervention. Together these convergent translational findings suggest altered local inhibitory networks in FXS pathophysiology can be evaluated using electrophysiology, and the findings may be predictive of clinical/behavioral pathologies relevant to drug development and testing.

The current study aimed to replicate previous EEG/ERP results in a larger sample of FXS patients from a different data collection site using different EEG equipment. The larger sample also enabled evaluation of gender differences in these phenotypes. In our previous preliminary study, clinical evaluation to establish correlation with electrophysiology was modest. In the current study, considerably more clinical data was collected to better establish the relevance of traditional electrophysiological measures with psychological and behavioral measures. We hypothesized that gamma measures would largely replicate in the new sample, be associated with both sensory and behavioral clinical measures, and be robust to reasonable levels of variability introduced by larger-scale data collection and analysis efforts. We further predicted that females with FXS would show reduced EEG/ERP abnormalities relative to males with FXS, consistent with reduced clinical/behavioral impairment in the majority of females with FXS.

## Materials and Methods

### Participants

Thirty-eight adolescents and adults with full mutation FXS [Mean (M) age = 25.5, standard deviation (SD) = 10.1; age range 10–53; 13 females] and 40 age- and sex-matched typically developing controls (M age = 27.7, SD = 12.1; age range 12–57; 17 females) participated in the study (Table 1). Most participants completed both habituation and chirp EEG tasks, but see Table 1 for exact demographic breakdown per task. Groups did not differ on proportion of each sex either overall (chi square = 0.57, *p* = 0.45), for the habituation task (chi square = 0.07, *p* = 0.79) or for the chirp task (chi square = 0.44, *p* = 0.51). Typically developing controls (TDC) had no known prior diagnosis or treatment for neuropsychiatric illness (reported via clinical history interview with parent or participant as appropriate). Exclusion criteria included history of seizures and current use of medications with known EEG effects, including anticonvulsant medications and benzodiazepines. Five patients were receiving atypical antipsychotics, 8 antidepressants, 8 both antipsychotics and antidepressants all on a stable dose for at least 4 weeks (see Supplementary Table 1 for a complete list). While medication effects cannot be ruled out, removing patients based on presence of commonly prescribed psychiatric medications would produce a sample that is non-representative of the FXS population. Our previous work and other EEG studies of these drugs in psychiatric research suggest they do not have significant confounding effects on electrophysiology as measured in the current study (Mitra et al., 2015; Ahnaou et al., 2016; Clementz et al., 2016; Ethridge et al., 2016, 2017); we also did not find any significant differences between medicated and non-medicated patients on any of the EEG variables studied here.

**TABLE 1:** Participant characteristics.

**FXS *n* = 38 (13 females)**				**Controls *n* = 40 (17 females)**				
	**Mean**	**Std. Dev.**	**Range**		**Mean**	**Std. Dev.**	**Range**	***t* Statistic (df)**
**Total sample**								
Age	25.5	10.1	10 to 53	Age	27.7	12.1	12 to 57	*t*(76) = 0.9, *p* = 0.37
Full scale IQ	60.3	20.9	47 to 115	Full Scale IQ	103.8	10.4	85 to 124	*t*(73) = 11.5, *p* < 0.001
Verbal Z	–3.0	1.9	−6.5 to 0.2	Verbal Z	0.14	0.71	−1.4 to 2	*t*(71) = 9.4, *p* < 0.001
Non-verbal Z	–4.6	2.4	−8.6 to −0.4	Non-verbal Z	0.21	0.76	−1.1 to 1.8	*t*(71) = 11.2, *p* < 0.001
Deviation IQ	41.7	28.9	−10.8 to 94.1	Deviation IQ	102.7	8.4	88.9 to 120.8	*t*(71) = 11.9, *p* < 0.001
SCQ	14.0	7.9	1 to 29	SCQ	2.2	2.4	0 to 8	*t*(63) = 8.0, *p* < 0.001

**FXS *n* = 30 (12 females)**				**Controls *n* = 37 (16 females)**				
	**Mean**	**Std. Dev.**	**Range**		**Mean**	**Std. Dev.**	**Range**	***t* Statistic (df)**

**Participants completing habituation task**								
Age	25.7	10.5	13 to 53	Age	26.8	11.9	12 to 45	*t*(65) = 0.4, *p* = 0.69
Full scale IQ	62.4	21.6	47 to 115	Full scale IQ	103.1	9.9	85 to 124	*t*(62) = 10.1, *p* < 0.001
Verbal Z	–2.8	1.8	−6.5 to −0.3	Verbal Z	0.1	0.6	−1.4 to 1.5	*t*(60) = 8.9, *p* < 0.001
Non-verbal Z	–4.6	2.6	−8.6 to −0.4	Non-verbal Z	0.2	0.8	−1.1 to 1.8	*t*(60) = 10.3, *p* < 0.001
Deviation IQ	43.7	30.2	−10.8 to 94.1	Deviation IQ	102.1	8.1	88.9 to 120.8	*t*(60) = 11.1, *p* < 0.001
SCQ	13.8	8.1	1 to 29	SCQ	2.3	2.4	0 to 8	*t*(54) = 7.7, *p* < 0.001

**FXS *n* = 36 (13 females)**				**Controls *n* = 39 (17 females)**				
	**Mean**	**Std. Dev.**	**Range**		**Mean**	**Std. Dev.**	**Range**	***t* Statistic (df)**

**Participants completing chirp task**								
Age	25.4	10.2	10 to 53	Age	27.9	12.2	12 to 57	*t*(73) = 0.9, *p* = 0.33
Full scale IQ	60.7	20.4	47 to 115	Full scale IQ	104.2	10.2	85 to 124	*t*(71) = 11.8, *p* < 0.001
Verbal Z	–3.0	1.9	−6.5 to 0.2	Verbal Z	0.2	0.7	−1.4 to 2.0	*t*(69) = 9.6, *p* < 0.001
Non-verbal Z	–4.5	2.4	−8.6 to −0.4	Non-verbal Z	0.2	0.7	−1.1 to 1.8	*t*(69) = 11.6, *p* < 0.001
Deviation IQ	42.4	29.1	−10.8 to 94.1	Deviation IQ	102.9	8.3	88.9 to 120.8	*t*(69) = 12.3, *p* < 0.001
SCQ	14.0	7.9	1 to 29	SCQ	2.2	2.4	0 to 8	*t*(62) = 8.2, *p* < 0.001

*SCQ, social and communication questionnaire.*

Primary caregivers completed the following clinical assessment measures for FXS patients: The Caregiver Report Adolescent and Adult Sensory Profile (Brown et al., 2001), the Social and Communication Questionnaire (SCQ; Rutter et al., 2003), Anxiety Depression and Mood Scale (ADAMS, Esbensen et al., 2003), Aberrant Behavior Checklist-Community (ABC-C, optimized for FXS, Sansone et al., 2012). We also administered the Woodcock-Johnson III Tests of Cognitive Abilities Auditory Attention subscale (McGrew and Woodcock, 2001), the Vineland Adaptive Behavior Scales (Sparrow et al., 2005) and the computerized Test of Attentional Performance for Children (KiTAP, Knox et al., 2012). IQ was assessed for both FXS and TDC with the Stanford-Binet Intelligence Scale 5th Ed. Abbreviated IQ (Roid, 2003) using deviation scores for calculating verbal and non-verbal IQ in the lower IQ range using the technique proposed by Sansone et al. (2014). Typically developing controls completed the SCQ, ADAMS, ABC-C, and KiTAP. All participants provided written informed consent (caregiver with assent or individual consent as appropriate) prior to participation, as approved by the Cincinnati Children’s Hospital Institutional Review Board.

### Procedure

#### Habituation Task

The auditory habituation stimulus consisted of 150 stimulus trains of four 50 ms duration white noise bursts separated by 500 ms inter-stimulus intervals. Each stimulus train was separated by a 4000 ms inter-trial interval. Habituation in this task is characterized as the change in ERP amplitude for each repetition in a stimulus train compared to the ERP amplitude to the initial stimulus in a train (e.g., initial N1 to 2nd N1, 3rd N1, and 4th N1).

#### Chirp Task

The auditory chirp stimulus consisted of a white noise burst carrier stimulus amplitude modulated by a sinusoid linearly increasing in frequency from 0 to 100 Hz over 2000 ms (16). Chirp stimuli were presented 200 times each separated by an inter-trial interval randomly jittered between 1500 and 2000 ms. For both EEG tasks, stimuli were delivered at 65 db SPL through headphones. Participants watched a silent movie during testing to facilitate compliance with testing procedures as in prior studies (Ethridge et al., 2016, 2017).

### ERP Recording

EEG was continuously recorded and digitized at 1000 Hz, filtered from 0.01 to 200 Hz, referenced to Cz, and amplified 10,000x using a 128 channel saline-based Electrical Geodesics system (EGI, Eugene, Oregon) with sensors placed approximately according to the International 10/10 system (42% of sensors in 128 channel EGI Hydrocel nets have 10–10 equivalents, while an additional 42% are within a 2 cm tolerance; Chatrian, 1985; Luu and Ferree, 2005).

### EEG Analysis

Raw data were visually inspected offline. Bad sensors were interpolated (no more than 5% per subject, no more than two adjacent, 90% of participants had no sensors interpolated within the 23 channels used in the final analyses) using spherical spline interpolation implemented in BESA 6.1 (MEGIS Software, Grafelfing, Germany). Data were digitally filtered from 0.5 to 120 Hz (12 and 24 db/octave roll-off, respectively; zero-phase; 60 Hz notch). Eye movement, cardiac, and muscle movement artifacts were removed blind to participant group using independent components analysis (ICA; Infomax) implemented in EEGLAB (Delorme and Makeig, 2004) using Matlab (The Mathworks, Natick, MA, United States). Segments of data with large amounts of movement artifact were removed prior to ICA to facilitate algorithm convergence. For both tasks, data were then transformed to average reference and epoched into 3250 ms trials (−500 to 2750 ms). For ERP analyses, data were averaged across trials and baseline-corrected using the 500 ms pre-stimulus period. Any trial with post-ICA amplitude exceeding 120 μV was considered residual artifact and removed prior to averaging. ERP averages for the habituation task were then low-pass filtered at 40 Hz for ERP analyses, while chirp averages and single trial power data for both tasks were retained at a low-pass filter of 120 Hz. Number of valid trials retained after artifact correction was higher for controls compared to FXS for the habituation task (FXS *M* = 105.5, SD = 22.4; Control *M* = 119.6, SD = 20.2, *t*(66) = 2.7, *p* = 0.008) and the chirp task (FXS *M* = 128.7, SD = 34.8; Control *M* = 152.9, SD = 32.7, *t*(75) = 3.2, *p* = 0.002), therefore trial count was evaluated as a covariate for all analyses and retained when significant.

**FIGURE 1:**
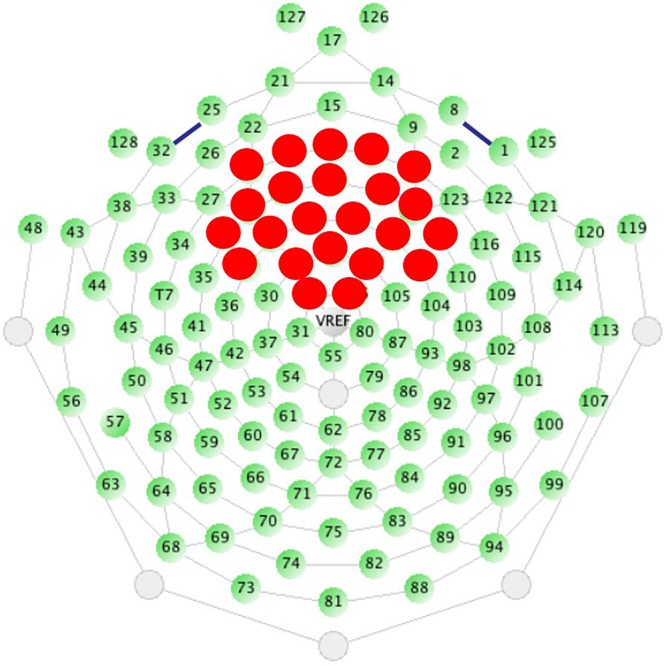
Sensor layout for the EGI 128 channel system with selected sensors analyzed highlighted in red. Sensors were selected based on the traditional location of the N1 ERP component, originating from auditory cortices, and from previous work.

Analyses in our previous studies used spatial principal components analysis (PCA) on the grand average ERP in order to create component weights using all sensors (Ethridge et al., 2016, 2017). However, the use of data-driven analyses is ultimately not scalable to clinical trials, which require *a priori* thresholds and defined regions of interest that can be calculated at the individual level without waiting for availability of large group averages for patient stratification. Therefore, we selected and averaged over 23 sensors distributed across the fronto-central scalp *a priori* based on the spatial distribution most consistent with previous literature capturing auditory cortex activity (Figure 1; Luck, 2014) and the peak of spatial activity from our previous PCA results (Ethridge et al., 2016, 2017). All analyses were conducted on data averaged over the same 23 sensors for both tasks.

For both tasks, un-baseline-corrected epoched single-trial data were analyzed in the time-frequency domain using Morlet wavelets with 1 Hz frequency step using a linearly increasing cycle length from 1 cycle at the lowest frequency (2 Hz) to 30 cycles at the highest (120 Hz). Single-trial power (STP) and inter-trial coherence (ITC) measures obtained from this method evaluated the amplitude of response at each frequency and how stable or phase-locked responses were to the auditory stimuli across trials, respectively (Delorme and Makeig, 2004). Raw ITC values were initially corrected for trial number by subtracting the critical r value, calculated as sqrt[-1/(number of trials)^∗^log(0.5)], for each subject based on trial count. STP and ITC values were averaged over trials for each individual and transformed into time-frequency plots down-sampled to 250 time-bins.

Single trial power was then baseline corrected using the pre-stimulus period, up to 50 ms prior to stimulus onset, to avoid windowing effects from stimulus onset-related activity. Subsequent analyses followed the same method as those done with non-baseline corrected single trial power.

### Statistical Analysis

For habituation ERP analyses, the waveform components of interest were the N1 and P2 components for the initial stimulus and each repeated stimulus in the stimulus train. N1 and P2 peaks were defined as the minimum and maximum amplitudes, respectively, in a time window centered on the grand average peak amplitude ± 40 ms. Amplitude and latency were calculated for each participant average at each peak. Separate mixed effects ANOVAs were calculated for amplitude and latency of each peak with the between subjects factors group (FXS, TDC) and gender (M,F) and within subjects factor stimulus repetition (initial stimulus, repetition 1, repetition 2, repetition 3). Differences in habituation of the N1 and P2 waveforms were calculated as the group by stimulus repetition interaction, indicating a difference between groups in the change in amplitude or latency across repetitions. Habituation was also calculated as percent change in N1 amplitude across repetitions, to match with our previous work.

For single-trial EEG analyses for both tasks, point-by-point two-tailed t-tests were used to calculate group differences across the time-frequency matrix. Time-frequency clustering techniques and Monte Carlo simulations controlled for multiple comparisons (Ethridge et al., 2012, 2017). To maintain a family-wise alpha of *p* < 0.01 (corrected for multiple comparisons), a minimum of three sequential time-bins and three adjacent frequencies were required to be significant at a nominal threshold of *p* < 0.05. Data were then averaged within each cluster to produce a single value for clinical correlations as well as univariate ANOVAs with fixed factors of group and gender. For all analyses, trial number and age were evaluated as covariates and retained in the model when significant. Effect sizes are reported as partial eta squared. Means presented are estimated marginal means.

Clinical correlations were examined with all significant variables. We also examined exploratory correlations between power in all frequency bands (theta, alpha, beta, gamma) and hypothesis-driven associations between gamma STP and gamma ITC. All correlations were conducted using Spearman’s rho. Clinical correlations and power band correlations were considered to be exploratory and hypothesis generating, and thus not corrected for multiple comparisons.

## Results

### Participant Demographics

There were no significant differences between FXS and TDC in age or proportion of gender. As expected with this clinical sample, FXS had significantly lower IQ scores and significantly higher number of autism-like symptoms on the SCQ than TDC (see Table 1 for detail).

### EEG

#### Habituation

**FIGURE 2:**
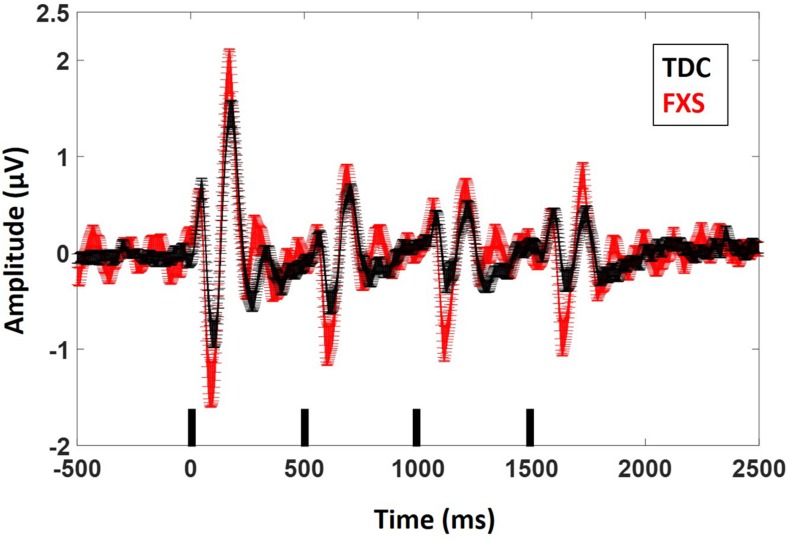
ERP average waveforms for FXS and TDC for the habituation task. Black marks along the x-axis indicate stimulus timing for the four stimuli in each train. Error bars indicate standard error of the mean. Note the increased N1 (negative peaks) and P2 (positive peaks) amplitude in FXS relative to TDC.

For N1 amplitude (Figure 2), there was a main effect of group, *F*(1,62) = 11.833, *p* = 0.001, ES = 0.16 indicating that FXS patients had larger N1 amplitudes (*M* = −1.47 μV, standard error (SE) = 0.13) than TDC (*M* = −0.85 μV, SE = 0.12). There was a marginal main effect of repetition *F*(3,186) = 2.5, *p* = 0.05, ES = 0.04 with a significant linear contrast *F*(1,62) = 6.07, *p* = 0.02, ES = 0.09. Pairwise comparisons to further examine this combination of effects indicated that N1 amplitude significantly (*p* < 0.001 for all repetitions) decreased across repetitions relative to the initial stimulus onset, but repetitions did not differ from each other, describing the plateau effect of subsequent repetitions: (N1 initial stimulus *M* = −1.60 μV, SE = 0.13; N1 repetition 1 *M* = −1.12 μV, SE = 0.10, N1 repetition 2 *M* = −0.99 μV, SE = 0.09, N1 repetition 3 *M* = −0.91 μV, SE = 0.09). There was no group by repetition effect, *F*(3,189) = 0.60, *p* = 0.61, ES = 0.01 suggesting that while FXS had larger N1 amplitudes overall, they did not habituate differently from TDC across repetitions. A repetition by gender effect *F*(3,186) = 2.67, *p* = 0.048, ES = 0.04 suggests that females plateau more strongly than males, who continue to decrease N1 amplitude across repetitions. Age was a significant covariate in the model *F*(1,62) = 7.13, *p* = 0.01, ES = 0.10, consistent with the literature that supports effects of age on N1 amplitude (Pang and Taylor, 2000), however age did not interact significantly with repetition effects (*p* = 0.21). There was no significant effect of trial count (*p* = 0.63). Similarly to our previous findings, we also quantified N1 habituation as percent change from N1 for the initial stimulus to each subsequent stimulus, however, FXS and TDC also did not differ on this comparison, *F*(2,124) = 0.61, *p* = 0.55, ES = 0.01 across all repetitions. Estimated marginal means for this comparison did show a large difference at the final repetition (percent reduction from initial stimulus to the last repetition in the train: FXS *M* = 25%, SE = 8%; TDC *M* = 51%, SE = 8%), suggesting that while it was not a strong effect, FXS may have shown decreased habituation relative to TDC by the end of the stimulus train. There were no differences between groups for N1 latency. There were no significant effects of age or trial count on percent change or N1 latency (*p*’s > 0.10).

Results were similar for P2 amplitude: there was a main effect of group, *F*(1,63) = 7.5, *p* = 0.008, ES = 0.11 indicating that FXS had larger P2 amplitudes (*M* = 1.37, SE = 0.10) than TDC (*M* = 0.99, SE = 0.09). There was a main effect of repetition, *F*(3,189) = 75.35, *p* < 0.001, ES = 0.55 indicating habituation of the P2 amplitude across repetitions (P2 initial stimulus *M* = 1.95, SE = 0.13; P2 repetition 1 *M* = 0.99, SE = 0.07, P2 repetition 2 *M* = 0.94, SE = 0.06, P2 repetition 3 *M* = 0.85, SE = 0.07). Again there were no group by repetition or gender effects, suggesting that although FXS patients had larger P2 amplitudes, they did not habituate differently. Age was not a significant covariate (*p* < 0.10). There was a main effect of group for P2 latency *F*(1,62) = 5.2, *p* = 0.03, ES = 0.08 such that FXS (*M* = 173.1 ms, SE = 2.47) had faster latencies than TDC (*M* = 180.7 ms, SE = 2.2). Age was a significant covariate *F*(1,62) = 4.09, *p* = 0.04, ES = 0.06. There were no significant effects of trial count on P2 amplitude or latency (*p*’s > 0.05).

**FIGURE 3:**
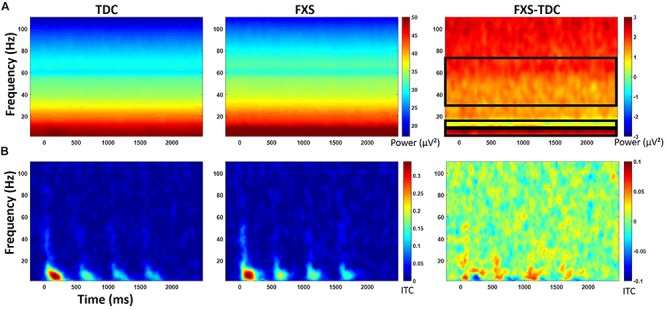
**(A)** Single trial power (STP) for TDC, FXS, and difference maps (FXS minus TDC) for the habituation task. **(B)** Inter-trial coherence (ITC) for TDC, FXS, and difference maps (FXS minus TDC) for the habituation task. Black boxes in the difference maps indicate clusters with significant group differences. Warmer colors (reds, yellows) in the difference maps (right column) indicate higher phase-locking or higher power for FXS and cooler colors (blues, greens) indicate higher values for TDC.

Point-by-point *t*-tests on non-baseline-corrected time-frequency plots for ITC and STP (corrected for multiple comparisons) revealed 3 time-frequency clusters with significant differences between FXS and TDC (Figure 3), all of which were in single trial power. Power values differences were largely consistent across the entire trial, including in the baseline, so values for each cluster were averaged across the entire trial and significant frequency range. For each of these comparisons, trial number was a significant covariate and retained in the analyses, but age was not a significant covariate. For theta (3–7 Hz) power, there was a main effect of group, *F*(1,62) = 9.12, *p* = 0.004, ES = 0.13 indicating that when correcting for number of trials, FXS (*M* = 50.9, SE = 0.44) showed higher theta power than TDC (*M* = 49.1, SE = 0.39). There were no gender effects on theta power. For alpha (8–12 Hz) power, however, there was no main effect of group, but a group by gender interaction, *F*(1,62) = 4.81, *p* = 0.03, ES = 0.07. FXS females (*M* = 49.2, SE = 0.82) showed higher alpha power than TDC females (*M* = 46.5, SE = 0.75) while FXS males (*M* = 48.07, SE = 0.69) and TDC males did not differ (*M* = 48.46, SE = 0.61). For gamma (31–70 Hz) power across the entire trial, there was a marginal effect of group, *F*(1,62) = 3.6, *p* = 0.06, ES = 0.05 indicating that FXS patients (*M* = 33.6, SE = 0.42) had marginally higher gamma power than TDC (*M* = 32.5, SE = 0.38). While there was a main effect of gender, *F*(1,62) = 5.63, *p* = 0.02, ES = 0.08 (males have more power than females), there was no interaction between group and gender.

For baseline-corrected single-trial power, FXS showed increased power in the beta/low gamma range (23–33 Hz) during stimulus onset for the initial stimulus only, *F*(1,63) = 10.97, *p* = 0.002, ES = 0.15. There were no effects of age, trial count, or gender on this comparison.

#### Chirp

**FIGURE 4:**
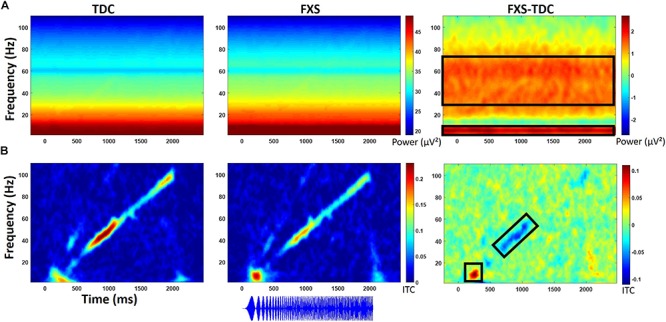
**(A)** Single trial power (STP) for TDC, FXS, and difference maps (FXS minus TDC) for the chirp task. **(B)** Inter-trial coherence (ITC) for TDC, FXS, and difference maps (FXS minus TDC) for the chirp task. Black boxes in the difference maps indicate clusters with significant group differences. Warmer colors (reds, yellows) in the difference maps (right column) indicate higher phase-locking or higher power for FXS and cooler colors (blues, greens) indicate higher values for TDC. Chirp stimulus schematic is represented bottom center relative to x-axis timing.

Point-by-point *t*-tests on time-frequency plots for ITC and STP (corrected for multiple comparisons) revealed 4 time-frequency clusters with significant differences between FXS and TDC (Figure 4). There was a main effect of group for alpha band (6–13 Hz) phase-locking (ITC) to the onset of the stimulus (92–308 ms post-stimulus), *F*(1,70) = 7.12, *p* = 0.009, ES = 0.09 indicating that FXS (*M* = 0.14, SE = 0.01) showed stronger phase-locking to the stimulus onset than did TDC (*M* = 0.09, SE = 0.01), consistent with habituation findings of increased ERP amplitude to auditory stimuli. Age was a significant covariate *F*(1,70) = 5.55, *p* = 0.02, ES = 0.07. There was a main effect of group for phase-locking (ITC) to the chirp stimulus (676–1066 ms post-stimulus, while the stimulus was in the low gamma oscillatory range) in the low gamma (31–57 Hz) band, *F*(1,71) = 5.65, *p* = 0.02, ES = 0.07 indicating that FXS (*M* = 0.11, SE = 0.01) were less able to lock in to the chirp oscillatory stimulus than TDC (*M* = 0.15, SE = 0.01). There was also a group by gender interaction, *F*(1,71) = 5.00, *p* = 0.03, ES = 0.07 indicating that while both males and females with FXS had lower phase-locking values, FXS females (*M* = 0.13, SE = 0.02) were more similar to both TDC females (*M* = 0.14, SE = 0.02) and TDC males (*M* = 0.16, SE = 0.02) than were FXS males (*M* = 0.08, SE = 0.02). Age was not a significant covariate (*p* = 0.97).

Gender effects were also found for ongoing (entire trial) theta power (3–7 Hz) during the chirp stimulus. First, there was a main effect of group, *F*(1,70) = 8.39, *p* = 0.005, ES = 0.11 indicating that FXS (*M* = 50.68, SE = 0.43) had higher theta (3–7 Hz) power than TDC (*M* = 48.93, SE = 0.40). There was also a group by gender interaction, *F*(1,70) = 5.74, *p* = 0.02, ES = 0.08. FXS females (*M* = 51.33, SE = 0.67) showed more theta power than TDC females (*M* = 48.19, SE = 0.58) while FXS (*M* = 50.03, SE = 0.53) and TDC (*M* = 49.65, SE = 0.52) males did not differ. For theta power, trial number was a significant covariate *F*(1,70) = 7.1, *p* = 0.01, ES = 0.09, and was retained in the analyses but age was not (*p* = 0.74).

For ongoing (entire trial) gamma (31–70 Hz) power, there was a main effect of group, *F*(1,71) = 7.4, *p* = 0.008, ES = 0.09 indicating that FXS (*M* = 33.58, SE = 0.32) showed more gamma power than TDC (*M* = 32.41, SE = 0.29). While there was a main effect of gender on gamma power, *F*(1,71) = 6.21, *p* = 0.02, ES = 0.08 (males have more gamma power than females in general), there was no interaction between gender and group. Age was not a significant covariate (*p* = 0.07).

**FIGURE 5:**
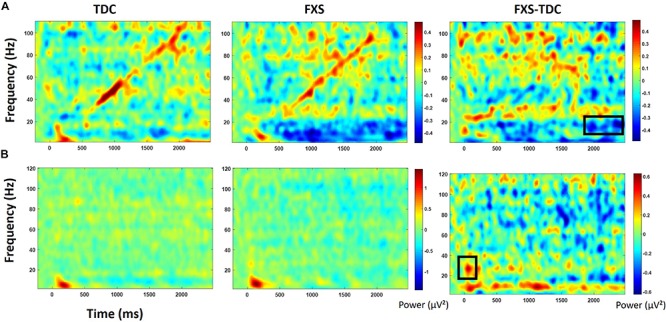
Baseline corrected single trial power for the chirp task **(A)** and the habituation task **(B)** for TDC, FXS, and difference maps (FXS minus TDC). Black boxes in the difference maps indicate clusters with significant group differences. Warmer colors (reds, yellows) in the difference maps (right column) indicate higher power for FXS and cooler colors (blues, greens) indicate higher values for TDC.

For baseline-corrected single trial power (Figure 5), FXS showed a decrease in alpha/beta power (11–20 Hz) which became significantly different from TDC during the time period surrounding stimulus offset (∼2000 to 2500 ms), *F*(1,71) = 15.44, *p* < 0.001, ES = 0.18. There were no effects of age, trial count, or gender on this group difference.

### Gamma Power and Phase-Locking

For the chirp stimulus, increased gamma single trial power across the entire trial was correlated with decreased gamma phase-locking to the chirp stimulus for TDC (rho = −0.34, *p* = 0.04). While the effect was in the same direction for FXS, it was not significant (rho = −0.22, *p* = 0.21). Gamma and theta power were also correlated for both groups (TDC rho = 0.34, *p* = 0.04; FXS rho = 0.35, *p* = 0.03) for the chirp task but were marginal for habituation. Gamma power was also correlated with beta power (13–30 Hz) for both groups (TDC rho = 0.61, *p* < 0.001; FXS rho = 0.49, *p* = 0.002), but while gamma power was also correlated with alpha power in TDC (rho = 0.50, *p* = 0.001), it was not for FXS (rho = 0.23, *p* = 0.18). Measures which were captured in both tasks (single trial power for gamma and theta bands) showed good correlation across tasks for both FXS and TDC. Gamma power (TD rho = 0.71, *p* < 0.001, FXS rho = 0.76, *p* < 0.001), and theta power (TD rho = 0.67, *p* < 0.001, FXS rho = 0.71, *p* < 0.001) both showed strong correlation between chirp and habituation tasks, suggesting good fidelity and test-retest reliability not just in these measures but also in the multiple-user blinded data processing approach utilized for this study.

### Exploratory Clinical Correlations

Significant correlations of electrophysiology data with clinical and behavioral measures in FXS participants are presented in Tables 2–7. Correlations are presented for all FXS patients first, then because gender and clinical/cognitive ability can be confounded in FXS, separated by gender. Increased gamma power and theta power were significantly related to a number of clinical ratings, including the ABC. Vineland subscale measures were also correlated with a number of spectral EEG measures across habituation and chirp tasks, primarily driven by correlations within female patients. Behavioral measures from the KiTAP were strongly correlated with spectral EEG measures, but most strongly correlated variables differed by gender.

**TABLE 2:** Significant clinical correlations for FXS patients.

**EEG measure**	**SCQ total score**	**Deviation IQ**	**ABC irritability**	**ABC stereotopy**	**ABC hyperactivity**	**ABC inappropriate Speech**	**WJIII**	**Vineland Comm**	**Vineland DL**	**Vineland Social**	**Vineland Comp**
**Habituation**											
N1 amplitude							−0.56^∗∗^				
P2 amplitude											
% habituation S1–S4	0.62^∗∗^								−0.48^∗^	−0.42^∗^	
STP theta								−0.53^∗∗^	−0.45^∗^	−0.43^∗^	−0.49^∗^
STP alpha								−0.43^∗^			
STP gamma		−0.43^∗^						−0.47^∗^	−0.44^∗^		−0.42^∗^
**Chirp**											
ITC stimulus onset							0.47^∗^				
ITC 40 Hz chirp	−0.53^∗∗^						0.49^∗∗^		0.36^∗^	0.38^∗^	
STP theta							0.46^∗^				
STP gamma		−0.54^∗∗^	0.37^∗^	0.42^∗^	0.39^∗^	0.41^∗^		−0.37^∗^	−0.41^∗^		

*Clinical variables with no significant correlations to EEG variables are not included. All correlations are Spearman’s rho. All correlations represent the FXS group only. Due to the negative amplitude of the N1 ERP, negative correlations should be viewed as increased N1 amplitude correlating with increased scores on the clinical/behavioral measure. Blank = N.S. ^∗^*p* < 0.05, ^∗∗^*p* < 0.01.*

**TABLE 3:** Significant clinical correlations for FXS patients – males only.

**EEG measure**	**SCQ total score**	**ABC social withdrawal**	**WJIII**	**Vineland DL**	**Vineland social**	**Vineland Comp**
**Habituation**						
N1 amplitude	0.63^∗^		−0.82^∗∗^			
P2 amplitude		−0.52^∗^				
% habituation S1–S4	0.77^∗∗^			−0.69^∗∗^	−0.61^∗∗^	−0.48^∗^
STP theta						
STP alpha						
STP gamma						
**Chirp**						
ITC stimulus onset			0.49^∗^			
ITC 40 Hz chirp	−0.44^∗^		0.47^∗^			
STP theta			0.53^∗^			
STP gamma						

*Clinical variables with no significant correlations to EEG variables are not included. All correlations are Spearman’s rho. All correlations represent the FXS group only. Due to the negative amplitude of the N1 ERP, negative correlations should be viewed as increased N1 amplitude correlating with increased scores on the clinical/behavioral measure. Blank = N.S. ^∗^*p* < 0.05, ^∗∗^*p* < 0.01.*

**TABLE 4:** Significant clinical correlations for FXS patients – females only.

**EEG measure**	**SCQ total score**	**Deviation IQ**	**ABC irritability**	**ABC social avoidance**	**Vineland comm**	**Vineland DL**	**Vineland social**	**Vineland comp**
**Habituation**								
N1 amplitude					0.71^∗^			0.75^∗^
P2 amplitude		−0.70^∗^	0.74^∗^		−0.84^∗∗^	−0.73^∗^	−0.73^∗^	−0.75^∗^
% habituation S1 to S4								
STP theta					−0.70^∗^			
STP alpha								
STP gamma		−0.71^∗^			−0.86^∗∗^	−0.69^∗^		−0.75^∗^
**Chirp**								
ITC stimulus onset								
ITC 40 Hz chirp	−0.77^∗^			−0.67^∗^				
STP theta								
STP gamma		−0.80^∗∗^						

*Clinical variables with no significant correlations to EEG variables are not included. All correlations are Spearman’s rho. All correlations represent the FXS group only. Due to the negative amplitude of the N1 ERP, negative correlations should be viewed as increased N1 amplitude correlating with increased scores on the clinical/behavioral measure. Blank = N.S. ^∗^*p* < 0.05, ^∗∗^*p* < 0.01.*

**TABLE 5:** Significant behavioral correlations for FXS patients.

**EEG measure**	**Distractor correct**	**Distractor errors**	**No distractor correct**	**Distract total correct**	**Flex correct**	**Go-NoGo errors**
**Habituation**						
N1 amplitude						
P2 amplitude						
% habituation S1–S4						
STP theta						
STP alpha						
STP gamma	−0.39^∗^			−0.39^∗^		
**Chirp**						
ITC stimulus onset		0.37^∗^				
ITC 40 Hz chirp					0.43^∗^	
STP theta						−0.39^∗^
STP gamma	−0.44^∗^		−0.38^∗^	−0.44^∗^		

*Clinical variables with no significant correlations to EEG variables are not included. All correlations are Spearman’s rho. All correlations represent the FXS group only. Due to the negative amplitude of the N1 ERP, negative correlations should be viewed as increased N1 amplitude correlating with increased scores on the clinical/behavioral measure. Blank = N.S. ^∗^*p* < 0.05, ^∗∗^*p* < 0.01.*

**TABLE 6:** Significant behavioral correlations for FXS patients – males only.

**EEG measure**	**Distract total correct**	**Flex correct**	**Go-NoGo correct**
**Habituation**			
N1 amplitude			−0.59^∗^
P2 amplitude			
% habituation S1–S4			
STP theta			
STP alpha			
STP gamma			
**Chirp**			
ITC stimulus onset			
ITC 40 Hz chirp		0.52^∗^	
STP theta			
STP gamma	−0.45^∗^		

*Clinical variables with no significant correlations to EEG variables are not included. All correlations are Spearman’s rho. All correlations represent the FXS group only. Due to the negative amplitude of the N1 ERP, negative correlations should be viewed as increased N1 amplitude correlating with increased scores on the clinical/behavioral measure. Blank = N.S. ^∗^*p* < 0.05, ^∗∗^*p* < 0.01.*

**TABLE 7:** Significant behavioral correlations for FXS patients – females only.

**EEG measure**	**Flex median**	**Flex correct**
**Habituation**		
N1 amplitude		
P2 amplitude	0.64^∗^	
% habituation S1 to S4		
STP theta		
STP alpha		
STP gamma		−0.63^∗^
**Chirp**		
ITC stimulus onset		
ITC 40 Hz chirp		
STP theta		
STP gamma		

*EEG measures with no significant correlations to clinical variables are not included. All correlations are Spearman’s rho. All correlations represent the FXS group only. Due to the negative amplitude of the N1 ERP, negative correlations should be viewed as increased N1 amplitude correlating with increased scores on the clinical/behavioral measure. Blank = N.S. ^∗^*p* < 0.05, ^∗∗^*p* < 0.01.*

## Discussion

The current study findings replicate and extend our previous findings of increased auditory N1 ERP amplitude, decreased gamma phase locking to a chirp stimulus, and increased gamma single trial power during the chirp task. We did not strongly replicate our prior finding of reduced habituation, although the general patterns seen across ERP repetitions is remarkably similar between our original study and the current. We utilized a larger sample in this study, enabling studies of effects in females who are underrepresented in the FXS research literature, and explored correlations of electrophysiological abnormalities with clinical and behavioral alterations associated with FXS. As this replication study was conducted using a new EEG system, different staff collecting and analyzing EEG data, these findings indicate both clinical scalability and clinical relevance of the electrophysiological findings.

Increased N1 ERP amplitude in FXS was replicated in this larger new sample, and we additionally found increased P2 ERP amplitude in FXS relative to TDC. In our previous work we found a marginal difference in N1 ERP amplitude between groups for the response to the initial stimulus, and significant amplitude differences for repeated stimuli, although ERP waveform plots suggested potentially larger amplitudes to all four stimuli. Here with more power to detect differences between groups, we show increased N1 amplitudes to all four stimuli in the stimulus train. We additionally found increased P2 amplitudes to all four stimuli in FXS, suggesting a generally hyper-excitable response throughout stimulus processing. Although we noted a possible *post hoc* difference between groups for habituation measured as percent change from the initial stimulus (S1) to the final stimulus (S4), we did not entirely replicate previous findings of decreased habituation of the N1 response across all repetitions, suggesting that the decrement in ERP amplitudes with repeated stimulation may not be a robust observation. One possibility for the lack of replication is, in the effort to increase translation between mouse and human, the change in stimulus from a 1000 Hz tone to a white noise burst. Increased stimulation of auditory cortices may have introduced the possibility of lateral inhibition effects, which can mimic habituation by decreasing ERP amplitudes for stimuli presented in close succession (Pantev et al., 2004). Rotschafer and Razak (2013) show broadened frequency tuning curves for individual neurons in auditory cortex in fmr1 KO mice, which may make FXS particularly sensitive to lateral inhibition effects. Alternatively, inhibitory interneuron dysfunction is characteristic of FXS (Cea-Del Rio and Huntsman, 2014), which may decrease lateral inhibition in FXS (Franco et al., 2017). Future work with masking stimuli is necessary to parse these effects and provide a mechanistic explanation for the differences in habituation effects found here.

The significantly hyper-excitable response (increased N1 amplitude) to repeated stimuli may still result in an increased attention to and lack of behavioral habituation to ongoing sounds (ability to “tune out”) in the environment. Indeed, for males, increased N1 amplitude was correlated with increased alertness and vigilance during Woodcock Johnson Auditory Attention Test. Interestingly, although FXS males and females did not differ on N1 amplitudes, the clinical relevance for increased N1 amplitude shows opposite effects based on gender: for males, increased N1 amplitude was associated with decreased scores on the SCQ, indicating fewer autism-like characteristics, and increased scores on the Woodcock-Johnson Tests. Indeed, individuals with idiopathic autism commonly show *reduced* ERP amplitudes relative to TDC (Jeste and Nelson, 2009), and in this case the hyper-excitable N1 response associated hyper-vigilance may improve ability to complete cognitive tests in individuals with intellectual disability. This is the first study known to date that links neural hyper-excitability to cognitive functioning in FXS. In females, however, increased N1 and P2 amplitudes were associated with decreased scores on the Vineland Adaptive Behavior Scales. This suggests that among females with FXS, neural hyper-excitability, and in turn hyper-vigilance, may impair functional abilities more broadly. However, it remains less clear whether this a gender-effect or due to the fact that females with FXS have more modest or no intellectual disabilities. Percent habituation also showed unusual gender effects, in that males with stronger habituation showed higher SCQ scores and lower Vineland scores, while females did not show correlations between these variables and habituation, suggesting that habituation and N1 amplitude are dissociable effects and may differentially impact clinical response. However, clinical correlations are presented as exploratory analyses, and further work designed to test specific clinical hypotheses is necessary to disentangle both gender and ERP effects on clinical variables.

A new finding for the habituation task was increased theta and alpha power in FXS relative to TDC. Our previous work showed similar trends (Ethridge et al., 2016) but with increased statistical power in the current study these group differences were statistically significant. Increased theta power has been commonly found for FXS in the resting EEG literature (Sabaratnam et al., 2001; Van der Molen and Van der Molen, 2013; Wang et al., 2017) and may reflect a compensatory response to reduced alpha-range thalamic modulation of high frequency cortical oscillations (Wang et al., 2017). Both theta and alpha oscillations may reflect thalamocortical modulation, but theta modulation is typically associated with longer range integration of cortical activity measured over anterior scalp and thus may be specialized for different functions relative to alpha, which is more commonly found to couple with gamma over posterior scalp (Canolty and Knight, 2010). The group by gender interaction for alpha power is interesting, in that FXS females do not show the reduced alpha (commonly associated with thalamic modulation) power that has previously been found in resting EEG literature with FXS patients (Van der Molen and Van der Molen, 2013; Wang et al., 2017); in fact, they showed increased alpha power relative to females without FXS.

Previous EEG literature in FXS has generally been underpowered to detect gender differences. Given that males and females both showed increased theta and gamma power and that they are correlated with each other, while alpha and gamma power are not, this finding may indicate a more complex relationship between high frequency (gamma) power abnormalities in FXS and thalamocortical modulation via alpha oscillations. This finding may be task-specific, since increased low frequency power in FXS was largely confined to the theta frequency band, where FXS females showed even more marked increases, even though both males and females with FXS showed increased gamma power. If the increased theta power does represent a compensatory effort to reduce high frequency activity as proposed previously, then this may indicate that female FXS patients may in part have higher functioning because of a more preserved ability to mobilize this response.

For the chirp task, we replicated previous findings of reduced ability to synchronize (phase-lock) high-frequency neural activity to the chirp stimulus. However, a group by gender interaction suggests that FXS females are considerably less impaired on this ability than FXS males. For both males and females, though, decreased phase locking to the chirp stimulus was associated with increased autism-like characteristics on the SCQ, similar to our previous findings (Ethridge et al., 2017). For males, cortical synchronization deficits were associated with reduced behavioral flexibility on the KiTAP, whereas the same deficits were associated with increased social problems in females. Cortical synchronization deficits were also associated with cognitive deficits on the Woodcock Johnson Auditory Attention Tests in males. Both males and females showed increased gamma power, although this finding was more robust in the chirp task, which may be due to stimulus-related effects, in that the chirp stimulus drives cortical oscillations in the gamma frequency range while the habituation task does not. For both tasks, increased gamma power was associated with decreased deviation IQ scores, suggesting a significant overall functional impairment associated with increased high-frequency neural “noise.” Indeed, gamma power correlated with increased distractibility on the KiTAP test and lower adaptive behavior scores on the Vineland, the latter mostly driven by a strong relationship between gamma power and adaptive behavior scores in females. Gamma power was also associated with an increase in severity of a number of behavioral problems and psychiatric issues, including irritability, stereotyped behaviors and speech, and hyperactivity. although these correlations were not significant when separated by gender, suggesting that they may correlate most strongly to differences in symptom severity that are commonly found to be associated with gender in FXS patients. These gender differences in clinical relevance for gamma power may also reflect the gender differences seen above in the theta and alpha bands, which may suggest a different effect of low frequency modulation of gamma power between genders. Future studies with specific clinical hypotheses will be necessary to examine the relationship between gender, symptom severity and EEG abnormalities.

The significant correlation between phase-locking abnormalities and increased non-specific gamma power was only partially replicated in this study, although the direction of the effect was the same as previously found (Ethridge et al., 2017). We have characterized increased gamma power as an increase in background neural “noise,” reducing overall signal-to-noise ratio (SNR) of sensory processing in auditory cortex. The overall reduction in strength for this comparison for both TD and FXS may be due to equipment differences between this study and the previous. Saline-based EEG systems like the one employed here typically have a higher impedance threshold and thus lower SNR, which although sensitive enough to capture overall group differences in gamma, may be less sensitive to capture smaller variations in gamma between individuals. Still, both gamma deficits were replicated in this sample, further highlighting deficits in neural synchronization related to local network excitation/inhibition balance supported by FXS translational rodent models and shown here to be related to core clinical deficits in FXS. In addition, the baseline-corrected single trial power findings from both tasks, of enhanced processing during the ERP at stimulus onset and then decreased or desynchronized low frequency activity during the chirp, suggest an increase in cortical “effort” accompanying processing the stimulus for FXS which may persist after stimulus offset and may indicate a potential homeostatic response to gamma processing deficits. FXS increase their gamma power relative to baseline similarly to TDC, however, the similar increase in gamma power riding on top of already significantly increased baseline power produces the group differences in un-baseline-corrected single trial gamma power and which is particularly evident during the chirp stimulus, which drives oscillatory networks at gamma frequencies. Similar findings in individuals with autism and their first degree relatives (Rojas et al., 2008; De Stefano et al., 2019) support a possible pathophysiological link in gamma power regulation across neurodevelopmental disorders.

Preclinical work in *Fmr1* knockout mice supports a mechanism for gamma abnormalities in decreased excitatory drive on fast-spiking inhibitory interneurons, resulting in increased and poorly synchronized pyramidal cell firing in the gamma range at rest and during stimulus processing (Gibson et al., 2008). Recent work suggests that intrinsic excitability in auditory cortex appears to be largely driven by synaptic activity between layers 2/3 and layer 5, which in contrast to previous work demonstrating network synchrony deficits, show a *hyper-synchrony* (Goswami et al., 2019). This hyper-synchronous response between cortical layers in the gamma frequency band may underlie the increased overall gamma power seen when neural activity is measured at the scalp, since increased gamma oscillatory activity is necessary to produce signals measurable at distant sources. This hyper-synchrony is also consistent with previous findings of poor stimulus-related synchrony, both at the slice level (Gibson et al., 2008) and in *in vivo* reductions in gamma phase-locking in both humans (seen here) and in Fmr1 KO mice (Lovelace et al., 2018). Phase-locking is driven by a resetting of the phase of ongoing oscillations in order to process an incoming stimulus. Intrinsically hyper-synchronous, hyper-excitable networks may be difficult to disrupt and modulate in order to produce accurate phase resetting, both reducing the signal processing ability and increasing the background “noise” of off-stimulus neural firing. Interestingly, increased ability to phase-reset and phase-lock gamma oscillations to the chirp stimulus was associated with increased behavioral flexibility on the KiTAP for both males and females with FXS, suggesting that local cortical flexibility measured at the sensory level may be related to higher-order cognitive flexibility.

Translational work with Fmr1 KO mice has reported similar findings to those reported here on both the habituation (Lovelace et al., 2016) and chirp tasks (Lovelace et al., 2018). An additional important step is necessary to fully validate these measures as functional outcome measure biomarkers and translate these findings for human clinical trials: scalability. This study addresses clinical scalability in a number of ways. First, we utilized a new group of subjects from a new clinic that recruits from a geographically distinct area from our previous work. Replication of our findings in this new sample suggests that these measures are robust to differences based on recruitment area, which is particularly important for FXS clinical trials which commonly utilize multi-site data collection strategies to increase patient numbers. Second, we used a different type of EEG equipment, in this case a saline-based EGI system, as compared to our previous work which used gel-based electrodes. Although we may have observed some minor system-related differences (see discussion above on signal to noise ratio), findings appear to be largely robust to system differences as well as reduced SNR associated with the EGI nets. This finding is important as saline-based EEG systems are becoming increasingly popular due to their ease of use, comfort, speed at which they can be applied, and reduced mess, all of which are important to reducing both patient and clinician burden as well as increasing the possibility of collecting useable data from more behaviorally impaired individuals. Equipment-invariance is also important due to the significant variation in existing equipment across clinics; purchasing identical EEG systems may not be financially feasible for large multi-site studies. Third, we used a white noise carrier sound for both habituation and chirp stimuli, which differs from the 1000 Hz carrier tone used in our previous studies. The white noise carrier sound stimulates a larger area of auditory cortex and produces a more robust neural response. Because it uses a wide range of frequencies rather than just one, the white noise sound can also be directly translated to rodent models without modification for hearing thresholds. The white noise sound is also less harsh-sounding to participants, and may allow for data collection in individuals with higher levels of sensory sensitivity. Our findings appear to be robust to these practical improvements in the stimulus properties, although see the discussion above regarding the habituation findings and white noise stimuli. Finally, we collected and analyzed the current dataset using a laboratory-based approach, with multiple research assistants and multiple data analysts all contributing to data collection, preprocessing, and screening. While all researchers involved were highly trained for reliability on their respective duties, this approach stills differs considerably from our previous work, which was collected and analyzed by a single individual. The laboratory approach is more consistent with large-scale studies with multiple research teams, and indicates that our findings are robust to the increase in error variance intrinsic to procedures with more decision-makers and decision points. We also analyzed the data slightly differently in order to produce *a priori* data cut-offs that can be utilized for individual data evaluation as well as interim data analyses, rather than data-driven approaches that can only be used once the entire dataset is collected. We used pre-defined sensors rather than data-driven topographic weights based on principle components analysis. Using predefined sensors also introduces the possibility of scaling the number of sensors necessary for data collection, reducing clinical burden. Taken together with similar findings in related disorders such as autism (Orekhova et al., 2008; Van Diessen et al., 2014; De Stefano et al., 2019), the replication of our previous work using different subjects, equipment, stimuli, and laboratory-based techniques point to ERP amplitude and gamma phase-locking and power as robust, clinically scalable measures that might be useful to predict or monitor drug response in large-scale multi-site clinical trials. Strong correlations between similar measures (gamma and theta power) between tasks also suggests test-retest reliability both of the measures and the laboratory-based data analysis strategies, another important factor in clinical trial readiness. While retest reliability for ongoing gamma (McFadden et al., 2014) and theta (Tan et al., 2015) power has been previously established, these findings support retest reliability of these measures specifically for FXS, a population for which increased response variability is sometimes a concern (The additional preclinical work showing replication of deficits and responsiveness to pharmaceutical intervention in FXS mouse models (Sinclair et al., 2017; Lovelace et al., 2018) suggests these measures may provide useful candidate biomarkers for treatment response in FXS. Specifically, both gamma phase-locking and power deficits show great promise as biologically grounded and functionally robust outcome measures in future clinical trials for FXS. Given the potential link to underlying pathophysiology, these biomarkers may also be relevant for other disorders with overlapping biological pathways and/or behavioral deficits that may ultimately arise from sensory processing abnormalities.

Despite these advances, some additional questions remain. Comparative work is still necessary to assess the specificity of our findings to FXS relative to other neurodevelopmental disabilities. Second, although we did not find differences between medicated and non-medicated patients, our sample is still not large enough to address the effects of individual medications; additionally, in correlational studies medication status may be confounded with symptom severity, necessitating targeted studies with pre-post designs. Excluding medicated patients can exclude a majority of FXS patients, particularly males and those with more severe clinical presentation, significantly impacting the representativeness of our data were they to be excluded from studies. So, we chose to retain patients in this study taking psychiatric medications commonly used to treat FXS patients that are not known to have significant impact on EEG measures obtained in the present study. Further, gamma abnormalities may be due to group differences in residual movement artifact due to common reduced behavioral compliance in FXS, however, preclinical studies of this question have found enhanced gamma power in fmr1 KO mice even during movement-free periods (Lovelace et al., 2018).

Preclinical models have provided a wealth of information relevant to understanding the genetic alteration resulting in FXS and its impacts on biochemical and local circuit function, but thus far the ability to translate these results into successful human clinical trials has been lacking. One reason for this disconnect may be the lack of translational biomarkers robust to both species differences and practical differences that may hinder reproducibility. The current study provides additional support for EEG-related neurophysiological measures as biomarkers by replicating previous findings in human data and linking them to a wider array of clinical features. Work is needed to link the EEG findings to molecular and network mechanisms in preclinical work, and also continue establishing their translational robustness.

## Data Availability Statement

The datasets used and/or analyzed during the current study available from the corresponding author on reasonable request.

## Ethics Statement

All participants provided written informed consent (caregiver with assent or individual consent as appropriate) prior to participation, as approved by the Cincinnati Children’s Hospital Institutional Review Board.

## Author Contributions

LE aided in study design, analyzed and interpreted the data, and wrote the manuscript. LD supervised EEG data management, quality control, and preprocessing. NW, KB, and MT contributed significantly to EEG data preprocessing. LS conducted the clinical assessments and supervised EEG data collection of all participants. JW aided in manuscript preparation and data interpretation. EP supervised EEG data collection and provided extensive contribution to manuscript preparation. CE supervised clinical assessment, participant recruitment, and contributed significantly to manuscript preparation and deviation IQ analyses. JS designed the study and contributed to all aspects of the research process, most significantly in data interpretation and manuscript preparation. All authors contributed substantially to the study, and read and approved the final version of the manuscript.

## Conflict of Interest

LE consults to OVID Therapeutics, Tetra Bio-Pharma, and Fulcrum Pharmaceuticals. EP has research grant support from StatKing. CE received current or past funding from Confluence Pharmaceuticals, Novartis, F. Hoffmann-La Roche Ltd., Seaside Therapeutics, Roivant Sciences, Inc., Fulcrum Therapeutics, Neuren Pharmaceuticals Ltd., Alcobra Pharmaceuticals, Neurotrope, Zynerba Pharmaceuticals, Inc., and Ovid Therapeutics Inc., to consult on trial design or development strategies and/or conduct clinical trials in FXS or other neurodevelopmental disorders. CE is additionally the inventor or co-inventor on several patents held by Cincinnati Children’s Hospital Medical Center or Indiana University School of Medicine describing methods of treatment in FXS or other neurodevelopmental disorders. The remaining authors declare that the research was conducted in the absence of any commercial or financial relationships that could be construed as a potential conflict of interest.

## Abbreviations

Db: decibels
EEG: electroencephalography
ERP: event-related potential
FXS: Fragile X Syndrome
GLU: glutamate
Hz: Hertz
ICA: independent components analysis
IQ: intelligence quotient
ITC: inter-trial coherence
KO: knockout
Ms: milliseconds
PCA: principal components analysis
PV+: parvalbumin positive
SCQ: social communication questionnaire
SNR: signal-to-noise ratio
STP: single trial power.
