# Supplementary material for: Auditory EEG Biomarkers in Fragile X Syndrome: Clinical Relevance
Source: Front Integr Neurosci. 2019 Oct 9;13:60. doi: 10.3389/fnint.2019.00060 (PMC6794497; doi:10.3389/fnint.2019.00060)
Supplement: Supplementary file 1 [file Table_1.DOCX]

Supplemental Materials.

Supplemental Table 1. Participant Medications

| Participant | Psychiatric Medications | Other Medications |
| --- | --- | --- |
| FXS |  |  |
| 1 | Adderall, Campral, Abilify | None |
| 2 | Concerta, fluoxetine | Zantac |
| 3 | Zyprexa | Zyrtec, Zantac |
| 4 | Campral, Concerta, Zoloft, Abilify | None |
| 5 | Adderall, Celexa, Intuniv | Melatonin |
| 6 | Vyvanse, Zoloft, Seroquel ER, Kapvay | Synthroid, melatonin |
| 7 | Zoloft, Campral, | Lipitor, carvedilol, fenofibrate, lisinopril |
| 8 | Vyvanse, Desyrel | None |
| 9 | Campral | None |
| 10 | Abilify, Celexa, Risperdal, Desryl | None |
| 11 | Invega, Abilify, Tenex, Zoloft, Thorazine | Metformin, Zantac |
| 12 | Prozac, Intuniv, Invega | None |
| 13 | Celexa, Abilify, Klonopin | Allegra |
| 14 | Abilify, Concerta, Celexa | Prilosec, Claritin |
| 15 | Celexa | None |
| 16 | Elevil, Abilify, Klonopin | Metformin, Melatonin |
| 17 | Concerta, Intuniv, Zoloft, Risperdal | Birth control |
| 18 | Baclofen, Prozac, Campral | Fenofibrate |
| 19 | Klonopin | None |
| 20 | Prozac | None |
| 21 | Vyvanse | None |
| 22 | Strattera, Remeron, Desryl | Birth control |
| 23 | Adderall, Celexa | Melatonin |
| 24 | Invega, Clonidine | None |
| 25 | Abilify | None |
| 26 | None | Birth control |
| 27 | None | Levothyroxin, selenium, multivitamin, kelp, fiber |
| 28 | None | Albuterol |
| 29 | None | Metformin |
| 30 | None | Birth Control |
| 31-38 | None | None |
| TDC |  |  |
| 1 | None | Zyrtec |
| 2 | None | Prevacid |
| 3 | None | Protonix |
| 4 | None | Birth control |
| 5 | None | Birth control |
| 6 | None | Flonase |
| 7 | None | Prilosec |
| 8 | None | Birth control |
| 9 | None | Birth control |
| 10 | None | Birth control |
| 11 | None | Zyrtec |
| 12 | None | Amoxicillin |
| 13 | None | Zyrtec |
| 14-40 | None | None |
